# What Is the Potential of *Daphnia* (Water Flea) Predation as a Means of Biological Suppression of *Prymnesium parvum* (Golden Algae) Blooms in Ecologically Relevant Conditions?

**DOI:** 10.3390/plants14121796

**Published:** 2025-06-11

**Authors:** Marta Galas, Marta Grabska, Maksymilian Zienkiewicz, Tomasz Krupnik

**Affiliations:** 1 Department of Biochemistry and Microbiology, Institute of Biology, Warsaw University of Life Sciences, Nowoursynowska 159, 02-776 Warsaw, Poland; marta_galas@sggw.edu.pl (M.G.); marta_grabska@sggw.edu.pl (M.G.); 2 Faculty of Biology, University of Warsaw, Miecznikowa 1, 01-096 Warsaw, Poland; mm.zienkiewicz@uw.edu.pl

**Keywords:** *Prymnesium parvum*, *Daphnia magna*, harmful algae blooms (HABs)

## Abstract

This study explores the interaction between *Prymnesium parvum* and *Daphnia magna* under low-salinity conditions. *P. parvum* showed reduced growth below 0.4 PSU and peaked at 1.0 PSU within the tested 0.2–1.0 PSU range. *D. magna*, exposed to *P. parvum* across 0.0–6.0 PSU, experienced increased mortality at 4.0 and 6.0 PSU, but tolerated 0.0–1.0 PSU well and grazed actively on *P. parvum* without significant vitality loss. This range reflects conditions observed in the Oder River during the 2022 fish die-off. The count of *P. parvum* cells did not vary significantly across the 0.2 to 1.0 PSU range of salinities in *D. magna* presence, except at 0.6 PSU. All daphnids survived even at *P. parvum* densities of 1 × 10^5^ cells/mL, though increasing algal concentrations reduced juvenile growth rates. Direct observation under a microscope confirmed algal ingestion. Toxin accumulation in cells and medium likely reduced grazing efficiency via allelopathic effects. The study assessed whether *D. magna* can tolerate prymnesins while maintaining feeding under varying salinities. Results suggest that *Daphnia magna* could act as a biological suppressor of golden algae under certain environmental conditions, though further work is needed to quantify grazing efficiency and prymnesins concentrations.

## 1. Introduction

The proliferation of harmful algae blooms (HABs) has raised significant concerns in aquatic ecosystems. *Prymnesium parvum* is a mixotrophic microalgae capable of producing potent toxins (prymnesins) that lead to mass fish kills (MFK) and ecological imbalances. In the summer of 2022, the Oder River, which flows through Poland and Germany, experienced a significant environmental disaster characterized by a massive loss of aquatic life, including fish, mussels, and snails. Investigations [1] identified the primary cause as a harmful algal bloom of *Prymnesium parvum*, commonly known as “golden algae”. The ecological consequences were severe, with an estimated 1000 metric tons of fish and numerous mussels and snails perishing. The disaster highlighted the vulnerability of freshwater ecosystems to HABs, especially when exacerbated by human activities such as industrial discharges [2] Of the available traditional methods, chemical and physical mitigation strategies often have unintended negative environmental impacts [3], necessitating the exploration of biological control methods. A promising approach is the use of *Daphnia* spp. (water fleas) as a means of controlling *P. parvum* blooms through predation during the early stages of golden algae proliferation.

### 1.1. Human-Engineered Application of Daphnia in Algal Bloom Control

*Daphnia* sp. as an obligate filter feeder is capable of filtering small particulate matter as well as microorganisms, contributing to the clarification of water in natural water ecosystems [4]. The Cladocera family, to which this particular *Daphnia* type belongs, are commonly spread in freshwater, mainly in lakes, although their presence is also being reported in river estuaries [5]. These properties have made *Daphnia* an object of interest in freshwater biological control methods.

The natural efficiency of *Daphnia* in controlling algae has allowed us to develop strategies to enhance their effectiveness through biomanipulation [6,7]; This involves deliberate efforts to increase the populations of *Daphnia* and their grazing pressure on the algae. An approach is the introduction of large-bodied *Daphnia* species into lakes and reservoirs, particularly in nutrient-rich (eutrophic) environments.

Another key strategy involves managing predator populations, as fish that feed on *Daphnia* (such as planktivorous fish) can limit their grazing effectiveness. By reducing the population of these fish or introducing predatory fish (such as pike) that feed on smaller fish, *Daphnia* populations can increase, leading to greater control of algal blooms [8]. Habitat improvement, such as improving water quality and increasing aquatic plant coverage, also helps to support *Daphnia* survival and reproduction.

The efficiency of *Daphnia* in controlling algal blooms has been demonstrated in various field studies. For example, in Lake Vesijärvi (Finland), a large-scale biomanipulation project involving fish removal and *Daphnia* stocking led to a significant reduction in algal biomass and improved water clarity [9]. Similarly, in Lake Mendota (USA), the introduction of large-bodied *Daphnia* combined with predator management resulted in reduced chlorophyll *a* concentrations and increased water transparency [10]. 

### 1.2. Adaptation of Prymnesium parvum to Low Salinity: Proliferation Potential in Freshwater Ecosystems

*P. parvum* can thrive at salinity levels ranging from 0.5 to 30 PSU (Practical Salinity Units), with optimal growth often observed around 15 PSU. However, different strains may exhibit varying salinity tolerances. Adaptation mechanisms of *P. parvum* to varying salinity involve physiological adjustments. Algae produce compounds like dimethylsulfoniopropionate (DMSP) and other polyols, which are believed to play a role in osmoregulation, helping the organism cope with changes in salinity [11].

Salinity not only affects the growth of *P. parvum*, but also its toxicity. Lower salinity levels can lead to decreased growth rates and reduced toxin production [12]. In contrast, optimal salinity conditions can enhance both growth and toxicity, contributing to the formation of harmful algal blooms. Despite the well-documented osmotic tolerance and efficient osmoregulatory mechanisms [13,14,15],; its cells appear to be highly sensitive to abrupt shifts in osmotic pressure. Rapid fluctuations in salinity cause the lysis of the *P*. *parvum* cell, leading to the release of its toxic metabolites, including prymnesins [16].

### 1.3. Nutritional Quality of the Culture of P. parvum Cells as Feed for Daphnia

A single-celled *Prymnesium parvum* is characterized by an ellipsoid or oval shape. Its cell dimensions typically range from 8 to 11 µm in length and 4 to 6 µm in width [17]. However, at extremes, the reported size range may exceed lengths between 6 and 12 µm and widths from 3.5 to 8 µm. These variations may be attributed to environmental factors, as research indicates that changes in water salinity can affect the cell volume of *P. parvum* [16]. *P. parvum* cells fall within the size range suitable for the ingestion of *Daphnia.* However, if the cell density is too high, the cells are too toxic, or they are nutritionally deficient, they may hinder *Daphnia*’s ability to graze efficiently [18,19]. Overcoming these limitations could be the key to utilizing *Daphnia* as an effective method of suppression of the bloom of *P. parvum* [20].

Our research aimed to evaluate whether *Daphnia magna* can suppress the proliferation of *Prymnesium parvum* under environmentally relevant salinity conditions. To this end, we first analyzed the growth of *P. parvum* across a selected salinity range. We assessed the toxicity of *P. parvum* to *D. magna* at salinities ranging from 0.0 to 6.0 PSU, with particular focus on the 0.0 to 1.0 PSU range. Mortality rates and ephippia production were monitored as indicators of *D. magna* stress responses. Subsequently, within the selected range of 0.0 to 1.0 PSU, we conducted grazing experiments to determine whether *D. magna* could reduce *P. parvum* cell densities. The suppression of *P. parvum* was quantified by assessing its cell growth across a range of salinity conditions in the presence of daphnids. To determine the cell concentration of *P. parvum* that could serve as a sole food source for *D. magna*, we performed juvenile growth rate assays. Under the same salinity condition (1.0 PSU), *D. magna* individuals were fed varying concentrations of *P. parvum*, and their growth was measured to identify a potentially toxic threshold of algal density.

Overall, our objective was to explore the potential application of *Daphnia magna* as a biological control agent to reduce *P. parvum* populations under salinity conditions that are ecologically relevant and tolerable for both species.

## 2. Materials and Methods

### 2.1. Prymnesium parvum Culture

The *Prymnesium parvum* strain CCAP 946/6 (CCMP708) was obtained from the Culture Collection of Algae and Protozoa (CCAP, Scotland, UK) and maintained in standard f/2 medium with 1.75 PSU sea salt. For low-salinity experiments, the sea salt concentration was reduced accordingly. Growth light was provided by fluorescent tubes or LED sources, and was set to 100 μmol·m^−2^·s^−1^ during the growth phase and 20 μmol·m^−2^·s^−1^ for precultures. Cultures were either grown in stationary conditions or agitated by bubbling with filtered compressed air (Millex^®^-FH air filter, 0.45 µm pores, Millipore, Burlington, MA, USA).

### 2.2. Daphnia magna Culture

The *Daphnia magna* clone used in this study was a monoculture established from a single female, following the method described in [21]’s study. The clone was obtained from the clone library of the Department of Biochemistry and Microbiology at the Warsaw University of Life Sciences, Poland. It originated from a city park pond in Warsaw, Poland (52°12′42.0″ N, 21°00′01.0″ E). Before and during the experiment, daphnids were kept under constant conditions, maintained by a climate cabinet (Plant Growth Chambers Sanyo MLR-350H, Moriguchi, Japan) at a temperature of 20 °C ± 0.5 °C and a summer photoperiod (16L:8D; 0.30 ± 2 μmol s^−1^ m^−2^). Prior to the experiment, the daphnids were fed daily with the green algae *Acutodesmus obliquus* at a non-limiting growth concentration of 1 mg total organic carbon (TOC) L^−1^, following Lampert [22].

*D. magna* were cultured in “Aachener Daphnien Medium” (ADaM) according to the protocol described by [23]. To standardize pre-experimental conditions and assess the specific effect of *Prymnesium parvum* on *D. magna*, daphnids were maintained in an increased salt concentration (1 g L^−1^) for two generations. For the experiments, third-clutch offspring from the second generation, cultured under these conditions, were used.

### 2.3. Acclimation of Daphnia magna to Experimental Salinity Conditions

Three adult females (7–10 days old) were transferred into 50 mL of ADaM medium with salinity adjusted to one of six levels: 0.0, 0.2, 1.0, 2.0, 4.0, or 6.0 PSU. Salinity was modified using sterile-filtered NaCl solutions, and each salinity treatment was performed in triplicate (n = 3), totaling 18 experimental units.

The animals were maintained under standard conditions (20 ± 1 °C; 16:8 h light:dark photoperiod) for 72 h. Mortality was assessed every 24 h, and the timing of ephippia (resting egg) production was recorded as an indicator of environmental stress.

### 2.4. Grazing of Daphnia magna on Prymnesium parvum Cells

Following the initial salinity tolerance trials, a second experiment was conducted to evaluate the grazing response of *Daphnia magna* on *Prymnesium parvum* under low-salinity conditions. Salinity levels were adjusted to 0.0, 0.2, 0.4, 0.6, 0.8, and 1.0 PSU, and each treatment was performed in triplicate (n = 3), resulting in 18 experimental units. Adult female *D. magna* (7–10 days old) were placed in 50 mL of ADaM medium supplemented with *P. parvum* as the sole food source. The algal density was standardized to 1.25 × 10⁴ cells/mL, corresponding to a non-limiting food concentration equivalent to 1 mg total organic carbon (TOC) per liter, as defined by Lampert [22]. The cell concentration of *P. parvum* was verified by optical density measurements at 800 nm (OD₈₀₀) using a UV-1900 spectrophotometer (Shimadzu, Kyoto, Japan) and by direct microscopic counting with a Neubauer hemocytometer (Paul Marienfeld GmbH & Co. KG, Lauda-Königshofen, Germany). Over the 72-h test period, mortality and ephippia production of D. magna were recorded at 24 h intervals.

### 2.5. Assessment of the Influence of Prymnesium parvum on Daphnia magna Juvenile Growth Rate

To evaluate the effect of *Prymnesium parvum* on the somatic growth of *Daphnia magna* neonates, a controlled growth rate experiment was conducted at a salinity of 1.0 g L^−1^ (1.0 PSU). Three neonates, all derived from the third clutch of the second laboratory-cultured generation maintained at 1.0 g L^−1^ salinity, were placed in 50 mL of ADaM medium with the same salinity. Each treatment condition was tested in triplicate (n = 3).

At the beginning of the experiment, all neonates were synchronized in age (<16 h old). They were fed *P. parvum* cells from whole culture suspensions (i.e., without centrifugation), thus containing both intact cells and extracellular exotoxins. The algal density was adjusted to provide a total organic carbon (TOC) concentration of 1.0 mg L^−1^, corresponding to the non-limiting food concentration for *Daphnia* growth [22]. Additional treatment groups were prepared using elevated *P. parvum* concentrations equivalent to 2.0, 4.0, and 6.0 mg TOC L^−1^ to assess dose-dependent effects.

Algal concentrations were verified using both optical density readings at 800 nm (OD₈₀₀) with a UV-1900 spectrophotometer (Shimadzu, Kyoto, Japan) and direct cell counts with a Neubauer hemocytometer (Paul Marienfeld GmbH & Co. KG, Lauda-Königshofen, Germany). A control group was included, in which *D. magna* were fed the green alga *Acutodesmus obliquus* at 1.0 mg TOC L^−1^.

The somatic growth rate of *D. magna* was determined by measuring body length at the start of the experiment and after four days of exposure, following the method [24]. Individuals were photographed using an optical microscope (Nexcope NE620; Ningbo Yongxin Optics Co., Ltd., Ningbo, China) equipped with a DLT-Cam PRO 8.3MP USB 3.0 camera (DLTA08300CMOSSEU3). Image analysis and length measurements were performed using DLT-Cam Viewer 3.7 software.

The specific growth rate (Gr) was calculated using the formula

$$Gr=\frac{ln[L_{t1}]-ln[L_{t0}]}{∆t}$$

where

L_t0_ is the length of the neonate at the start of the experiment (<16 h old),

L_t1_ is the length of the 4-day-old daphnid,

Δt is the time (in days) between the first and fourth day of the experiment.

The medium was replaced every two days, with an appropriate food supply provided.

### 2.6. Statistical Analysis

Data were analyzed using R software (version 4.0.3). The normality of data distribution was assessed with the Shapiro–Wilk test. For data following a normal distribution, an ANOVA was performed, followed by Tukey’s Honest Significant Difference (Tukey-HSD) post hoc test for multiple comparisons. For non-normally distributed data, the Kruskal–Wallis rank sum test was applied. In all tests, the significance level was set at α = 0.05.

## 3. Results

### 3.1. Growth Rates of Prymnesium parvum Cells in Selected Conditions of Salinity

**Figure 1:**
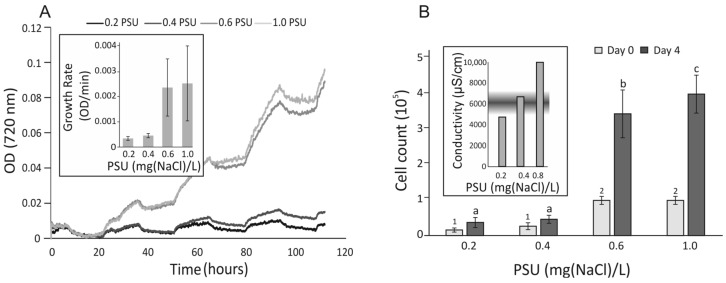
Growth curves of *Prymnesium parvum* cells under selected salinity conditions. (Panel (**A**)) Cell growth was continuously monitored in duplicate cultures across a salinity range of 0.2–1.0 PSU. (Inset (**A**)) Growth rates were calculated as the mean increase in cell density during the light phase. (Panel (**B**)) Cell counts were recorded before and after cultivation. Mean values are shown, and statistical analysis was performed using ANOVA followed by Tukey’s post hoc test in R. Statistically significant differences were annotated in groups with different low-case letters at the end of experiment and numbers at the beginning of the experiment. (Inset (**B**)) Conductivity values corresponding to the tested salinity range are shown, with the shaded area indicating the range associated with mass fish kill (MFK) events in the Oder River.

*Prymnesium parvum* cells were cultured in f/2 medium for 120 h (5 days) using a multicultivator (PSI, Drásov, Czechia) under a light intensity of 80–100 μmol⋅m^−2^⋅s^−1^ in a 12 h/12 h light/dark cycle, in duplicate (Figure 1A). The fastest growth was observed at salinities between 0.6 and 1.0 PSU. Growth rates (inset A) in this range were 3–3.5 times higher compared to the 0.2–0.6 PSU range. Cell counts (Panel B) in the higher salinity range (0.6–1.0 PSU) were 6–7 times greater than those in the lower range (0.2–0.4 PSU, *p* < 0.05). No significant differences in cell counts were observed within the low-salinity range. Although cell growth in this range increased ~1.5-fold, it was not statistically significant (*p* > 0.05). Conductivity under the tested salinity conditions was measured after the growth period (inset B). The tested salinity range overlapped with the conditions associated with mass fish kills (MFK) in the Oder River in 2022 [25].

### 3.2. Physiological Response of Daphnia magna to Prymnesium parvum

**Figure 2:**
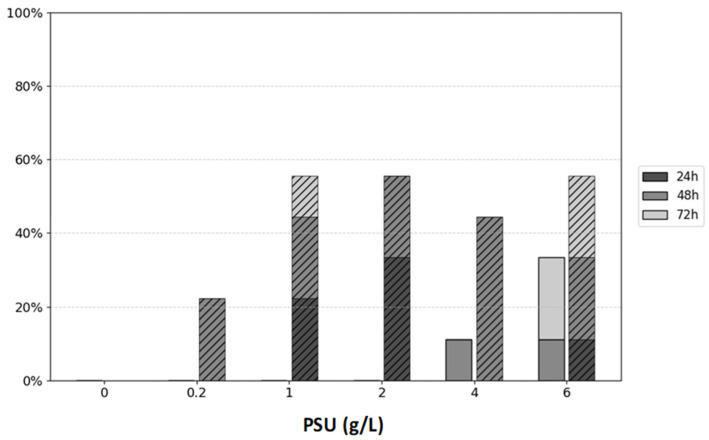
The figure represents *Daphnia’s* mortality (solid bars) and ephippia production (hatched bars) at three time points (24, 48 and 72 h) under different salt concentrations ranging within 0.0–6.0 PSU (g/L). Mortality and ephippia production are presented as percentages [%] of the total number of daphnids (9 individuals) in each variant.

To assess whether *P. parvum* poses a threat or causes mortality in obligate filter-feeding organisms such as daphnids, a *D. magna* clone culture was used in this study. For each experimental variant, three adult daphnids were used in triplicate, totaling nine daphnids per condition. *P. parvum* served as the sole food source under different salt concentrations for 72 h. The physiological responses of *D. magna* under these experimental conditions are summarized in Figure 2. The mortality of water fleas fed *P. parvum* was confirmed only in 4.0 and 6.0 PSU conditions. The highest mortality was noted in *D. magna* fed *P. parvum* under the 6.0 PSU condition. *D. magna* also showed an ecological response to stress in their environment through the production of ephippia. The fastest ephippia production was noted in 1.0, 2.0, 6.0 PSU. Interestingly, even in a proportionally low concentration of salt (0.2 PSU), ephippia occurrence under daphnid carapax was confirmed after 48 h.

### 3.3. Prymnesium parvum Cell Count in the Presence of Filter-Feeding Daphnia magna

**Figure 3:**
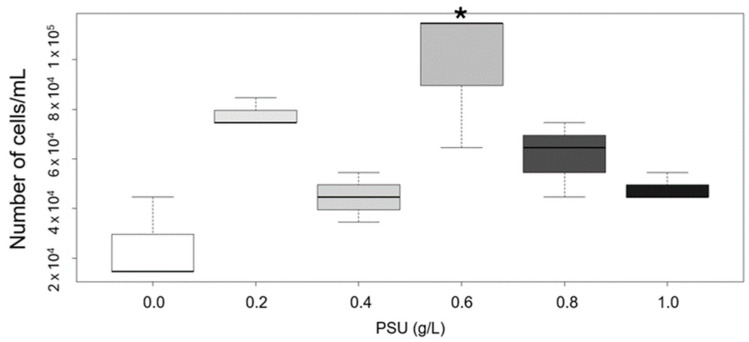
Difference between *Prymnesium parvum* cell count at the start and after 72 h in the presence of *Daphnia magna.* At the beginning of the experiment, the cell number of *P. parvum* used as the sole food source for daphnids was measured and adjusted to fulfill the non-limiting concentration of total organic carbon (TOC), which is 1 mg L^−1^. The evaluation of cell number showed that 1 mg TOC concentration is equal to 1.25 × 10^4^ cells/mL of *P. parvum* cells. For each variant, we used the same number of cells at the beginning and checked the cell number of *P. parvum* after 72 h of presence to filter feeding *D. magna* in different salinity concentrations. Change in cell number within that time is represented as boxplot. In each box, a central bold line represents the median. Whiskers in every box stand for minimal and maximal values. Asterisk indicate statistically significant difference between variants with different salinity in comparison to the control (1.0 PSU) (*p* < 0.01, one-way ANOVA and post hoc Tukey HSD). Each box represents *P. parvum* cells in the presence of *D. magna* at different salinity levels; white box—0.0 PSU, light gray—0.2 PSU, gray—0.4 PSU, dark gray—0.6 PSU, the darkest gray—0.8 PSU, and the black one—1.0 PSU, used as a control in this study.

Cytotoxic *Prymnesium parvum* (containing exotoxins) was used as the sole food source for *Daphnia magna*, provided at a non-limiting growth concentration of 1.0 mg total organic carbon (TOC) per liter of medium. As shown in Figure 3, over the course of three days, in the presence of obligate filter-feeding *D. magna*, *P. parvum* cell density tended to increase. The highest increase in *P. parvum* cells during presence of *D. magna* was noted for the 0.6 PSU concentration of salt.

A 1.0 PSU salt concentration was used as the control in this study to determine whether daphnids, which were primary adjusted to this salinity, could survive when *P. parvum* was provided as the sole food source. This salinity was also in the range within which *P. parvum* cells’ proliferation normally occurs. What is worth noticing is the fact that the experiment was conducted with a logarithmic increase in salt concentration, mimicking conditions observed during golden algae blooms. After 72 h, all daphnids remained alive under all the experimental conditions.

### 3.4. Impact of Prymnesium parvum on Daphnia magna Juvenile Growth Rate

**Figure 4:**
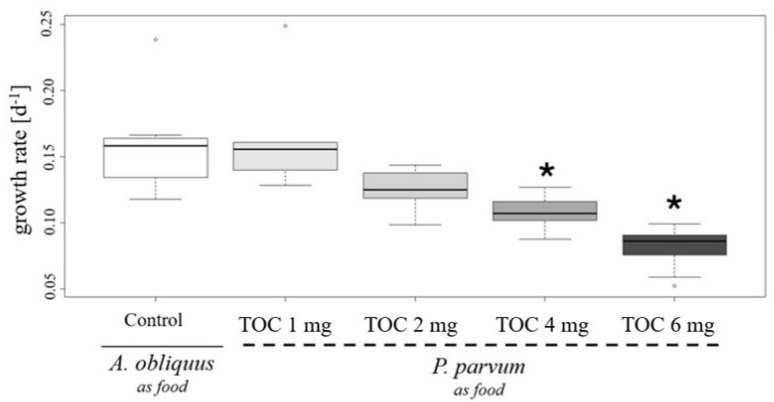
Change in growth rate of *D. magna* caused by *Prymnesium parvum* influence as a nutrition source. Three neonates, previously adapted to salt concentration of 1 g L^−1^, were used in the study, with three replicates per variant, resulting in nine individuals per variant. The study was conducted at a salinity of 1.0 PSU. *P. parvum* was provided as the sole food source for the neonates, with concentrations of 1.0 mg (light gray box), 2.0 mg (gray box), 4.0 mg (dark gray box), and 6.0 mg total organic carbon (TOC) per liter (darkest gray box). As the control, neonates were fed green algae *Acutodesmus obliquus* at a concentration of 1.0 mg TOC per liter, representing the minimal non-limiting growth concentration (white box). The evaluation of TOC concentration indicates that for *P. parvum*, 1 mg TOC is equal to 1.25 × 10^4^ cells/mL, and for *A. obliquus*, 1 mg TOC is represented by 9.85 × 10^5^ of cells/mL. The boxplot shows the growth rates over time. The bold central line in each box represents the median value, while the whiskers indicate the minimum and maximum values. Outliers are marked as circular points. Stars indicate statistically significant differences between variants with *P. parvum* as the food source compared to the control (*p* < 0.001), based on the non-parametric Kruskal–Wallis test and post hoc Tukey HSD.

One of the most significant vital parameters for *D. magna* is the somatic growth rate of juveniles [24]. The results shown in Figure 4 demonstrate that an increase in *P. parvum* cell density, when used as food for juvenile daphnids, leads to a decrease in their growth rate, as measured after four days of the study. During this study, all neonates survived in the presence of *P. parvum* cells. However, in the variant with 6 mg TOC L^−1^ from *P. parvum* cells, one juvenile daphnid became immobilized. These findings suggest that higher densities of *P. parvum* may disrupt certain aspects of *D. magna* development.

**Figure 5:**
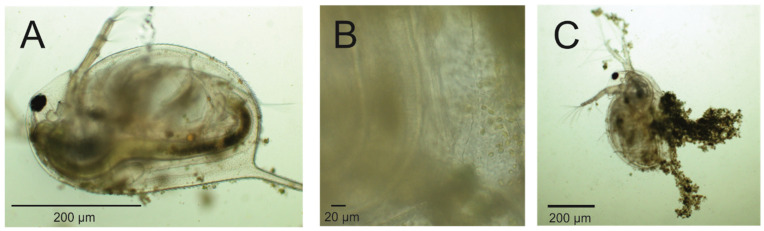
Interaction between *Daphnia magna* and *Prymnesium parvum* cells observed with an optical microscope. *Daphnia magna* was collected from suspension with 6 mg TOC per liter, which is equal to a density of 7.51 × 10^4^ cell/mL of *P. parvum*. The feeding behavior (i.e., rhythmic movements of the thoracic appendages) of the *Daphnia* specimen was observed under a microscope (Nexcope NE620). The presence of *Prymnesium parvum* cells within the gut of the specimen was confirmed (**A**,**B**), although an aggregation of algal cells around daphniid, resulting in reduced mobility, was also observed (**C**).

A single *Daphnia* specimen was isolated from a suspension containing *Prymnesium parvum* cells after 4 days of grazing for each image, where *P. parvum* cells were introduced for *Daphnia magna* at 6 mg L^−1^ TOC concentration in 1.0 PSU salinity. Characteristic filter-feeding behavior was observed (Panel A). The presence of *P. parvum* cells within the digestive tract of *Daphnia* (Panel B) confirms that *Daphnia* is capable of grazing on *P. parvum*. Additionally, *P. parvum* cells were clearly visible near the antennae and in the immediate surroundings, exhibiting their typical oval shape (8–10 µm in length) and green-yellow pigmentation (Panel B). Although visible in Panel C, the accumulation of *P. parvum* cells around the *D. magna* body may indicate that 6 mg L^−1^ TOC concentration of algae causes progressive proliferation of golden algae cells, followed by daphnid immobilization. This may partially disable *Daphnia* filtration, causing a limitation of the food source for daphnids, followed by further distractions in life parameters, which corresponds to the results obtained in neonates’ growth rate represented in Figure 5.

## 4. Discussion

### 4.1. Growth Conditions

Our results suggest that both *Prymnesium parvum* and *Daphnia magna* exhibit tolerance to the salinity levels selected for the experiment [12]. The salinity range of 0.2–1.0 PSU lies at the lower end of the tolerance spectrum for *P. parvum*, whose optimal range is reported to be between 5.0 and 20.0 PSU [26]. However, the selected range reflects conditions observed during the mass fish mortality event in the Oder River in 2022, where salinity levels, inferred from conductivity measurements (850–7000 µS/cm), ranged between 0.5 and 1.2 PSU [1,25].

The proliferation rate of *P. parvum* (Figure 1A) was highly dependent on salinity. Concentrations below 0.4 PSU were particularly inhibitory, reducing cell growth by approximately 3 to 3.5 times compared to 1.0 PSU. Cell density was also significantly reduced in conditions below 0.4 PSU (Figure 1B), whereas at 1.0 PSU, it reached approximately 4 × 10^5^ cells/mL—almost double the mean levels recorded in the Oder River in 2022 and comparable to the highest levels reported by Sobieraj and Metelski [25]

*Daphnia magna* generally does not tolerate salinities above 6.0 PSU, although this is a species-specific trait. In our study, *Daphnia* displayed sensitivity at 4.0 PSU, with one individual dying after 48 h, and at 6.0 PSU, where one individual died within 48 h and two other individuals died after 72 h (Figure 2). In the test presented in Figure 2, we evaluated the sensitivity of *Daphnia* fed with *P. parvum* in a 0.0 to 6.0 PSU range of salinity. Results demonstrate that salinity concentrations above 1.0 PSU are responsible for ephippia (resting egg) production by around 50% of daphnids used in the test. Ephippium egg production is an important part of daphnids’ ecological strategy to survive under unfavorable conditions and indicate their physiological status. Usually, ephippium egg formation takes place when seasonal changes appear, but it may also be caused by biotic, e.g., predator stress or abiotic stressors, e.g., the pH of water or toxins [21,27,28]. 

The *Daphnia magna* mortality experiment depicted in Figure 2 suggests that at higher salinity levels (6.0–10.0 PSU), *D. magna* may be unable to graze efficiently on *Prymnesium parvum* due to the combined effects of osmotic stress and exposure to algal toxins. These cumulative stressors likely exceed the physiological tolerance of *D. magna*, leading to increased mortality. In contrast, the lower salinity range of 0.0–1.0 PSU was well tolerated, with minimal adverse effects observed. This range aligns with ecologically relevant conditions where both *D. magna* and *P. parvum* are known to.

### 4.2. Prymnesium parvum Cell Count in the Presence of Filter-Feeding Daphnia magna

In the presence of preculture medium likely containing residual *P. parvum* toxins, increased *P. parvum* proliferation was observed. This could be explained by allelopathic effects that compromised *Daphnia* vitality. Interestingly, the highest growth rate of *P. parvum* was observed at 0.6 PSU. This salinity is just below a critical threshold [16], where osmotic stress induces cells to adopt an inflated, spherical morphology. We propose that conditions around 0.6 PSU may induce prymnesin production due to cellular stress, without exceeding the cell’s ability to maintain membrane integrity. At lower salinities (<0.6 PSU), elevated mortality may hinder toxin production and prevent the environmental accumulation of lethal prymnesins concentrations, allowing for more effective *Daphnia* grazing. Conversely, at higher salinities, osmotic conditions may favor *P. parvum* cell integrity and reduce toxin production [29], thereby allowing *Daphnia* for better grazing.

The susceptibility of *Daphnia* to *Prymnesium parvum* toxins was evaluated by exposing individuals to a suspension equivalent to 1 mg L^−1^ total organic carbon (TOC), corresponding to approximately 1.25 × 10^4^ cells/mL (Figure 3). However, in the grazing test (Figure 3), the observed susceptibility in our experiments was markedly lower than previously reported by Remmel et al. [30]; our test organisms survived at higher cell densities, corroborating the findings of the same study under specific conditions. These observations are more closely aligned with the findings of Cagle et al. [31], who also reported relatively low *Daphnia* mortality under high *P. parvum* cell densities.

In contrast, the actual toxicity of Oder River water during the harmful algal bloom (HAB) event was assessed on the day of peak bloom intensity using a *Daphnia* bioassay [32]. The results revealed acute toxicity, with *Daphnia* mortality exceeding 90% even after a fourfold dilution, within 24 and 48 h of exposure. These findings indicate that under bloom conditions, environmental samples may contain significantly higher levels or more bioavailable forms of prymnesins than those observed in laboratory-controlled cultures.

To verify the ingestion of *P. parvum* cells by *Daphnia*, microscopic examination was conducted. The presence of *P. parvum* cells within the digestive tracts of test animals was confirmed (Figure 5), providing direct evidence of active grazing under all tested conditions.

### 4.3. Life History Parameters

With a higher density of golden algae cells, corresponding to both 4 and 6 mg L^−1^ TOC concentrations, reduced growth rates of juvenile daphnids were noted in our study. Juvenile growth rate is an important parameter which evaluates *D. magna* life history. Reduced growth rate may be caused by many different factors, for example, chemicals, toxins, cyanobacteria presence, or worse quality and limited food [33]. As represented in Figure 5, Panel C, with higher cell density an accumulation of cells occurred which partially hindered daphnids in performing filtration, and might have probably caused worse food uptake, despite its higher accessibility. Another explanation is that with a higher density of *P. parvum* cells, a higher toxin concentration is noted, which causes a reduced growth rate of *D. magna* neonates.

When it comes to changes in water chemistry, phosphorus (P) and nitrogen (N) concentrations may also be impeded during harmful algal blooms. Both of those elements’ acquisitions are limitation factors, not only for *P. parvum* but also in *Daphnia* sp. physiology. During imbalanced stoichiometry in the N:P ratio in a high-density population of *P. parvum* cells, algae produce more toxins and chemical substances, which may exert an allelopathy effect of *Daphnia*, causing their higher mortality [31]. This may explain why, even with the high density of algae (6 mg L^−1^ TOC) in our study (Figure 5), daphnids survived during four days of investigation. Similarly to Cagle et al. [31]’s study, the *P. parvum* used in our experiment was cultured in a balanced N:P ratio before being introduced to *Daphnia*, and we did not obtain high mortality, especially in 0.2 to 1.0 PSU, as probably in these conditions the deleterious effects of toxins released from golden algae cells is low.

Thus, this study underlines the importance of freshwater ecosystems’ environmental monitoring, and the need to take action before HABs occur. We evaluated the usage of biological control methods by the application of *D. magna*, which may potentially help to reduce golden algae cells’ quantity. Nevertheless, this study indicates that action should take place before environmental catastrophes occur. In addition, interactions between the organisms described and presented here still demand further studies, both in the laboratory and environmental conditions.

## 5. Conclusions and Future Work

We have shown that *Daphnia magna* can coexist with and feed on *Prymnesium parvum* cells, potentially contributing to the suppression of algal blooms. Prymnesins in tested conditions do not appear to significantly hinder *Daphnia magna* growth, which contrasts with some previously published data. To address discrepancies in the literature, a more precise quantification of cell densities, salinity, and prymnesins concentrations is needed. Unfortunately, such analyses are constrained by the lack of standardized reference materials and the need for specialized analytical equipment. Further quantitative studies on *Daphnia*’s grazing efficiency are also warranted. We suggest that daphnids may survive at least the beginning of *P. parvum* blooms and could potentially serve as biological suppressors of harmful algal communities. As obligate filter feeders, they may partially consume *P. parvum* cells. However, higher daphnid densities, longer exposure times, and increased replication are necessary to draw definitive and statistically robust conclusions.

## Data Availability

The raw data supporting the conclusions of this article will be made available by the authors on request.
